# Increasing Complexity in Wireframe DNA Nanostructures

**DOI:** 10.3390/molecules25081823

**Published:** 2020-04-16

**Authors:** Petteri Piskunen, Sami Nummelin, Boxuan Shen, Mauri A. Kostiainen, Veikko Linko

**Affiliations:** 1 Biohybrid Materials, Department of Bioproducts and Biosystems, Aalto University, P.O. Box 16100, 00076 Aalto, Finland; petteri.piskunen@aalto.fi (P.P.); sami.nummelin@aalto.fi (S.N.); boxuan.shen@aalto.fi (B.S.); mauri.kostiainen@aalto.fi (M.A.K.); 2 HYBER Centre, Department of Applied Physics, Aalto University, P.O. Box 15100, 00076 Aalto, Finland

**Keywords:** DNA nanotechnology, DNA origami, self-assembly, computer-aided design, wireframe structures, meshing, algorithmic design, top-down, nanofabrication, biomaterials

## Abstract

Structural DNA nanotechnology has recently gained significant momentum, as diverse design tools for producing custom DNA shapes have become more and more accessible to numerous laboratories worldwide. Most commonly, researchers are employing a scaffolded DNA origami technique by “sculpting” a desired shape from a given lattice composed of packed adjacent DNA helices. Albeit relatively straightforward to implement, this approach contains its own apparent restrictions. First, the designs are limited to certain lattice types. Second, the long scaffold strand that runs through the entire structure has to be manually routed. Third, the technique does not support trouble-free fabrication of hollow single-layer structures that may have more favorable features and properties compared to objects with closely packed helices, especially in biological research such as drug delivery. In this focused review, we discuss the recent development of wireframe DNA nanostructures—methods relying on meshing and rendering DNA—that may overcome these obstacles. In addition, we describe each available technique and the possible shapes that can be generated. Overall, the remarkable evolution in wireframe DNA structure design methods has not only induced an increase in their complexity and thus expanded the prevalent shape space, but also already reached a state at which the whole design process of a chosen shape can be carried out automatically. We believe that by combining cost-effective biotechnological mass production of DNA strands with top-down processes that decrease human input in the design procedure to minimum, this progress will lead us to a new era of DNA nanotechnology with potential applications coming increasingly into view.

## 1. Introduction

In the early 1980s, the research field dubbed DNA nanotechnology was born along with the theoretically predicted, rationally designed objects composed of a few DNA strands [1] connected via Watson-Crick base pairing [2]. Ned Seeman, the pioneer of the field, postulated and later on experimentally demonstrated structures that were inspired by a Holliday junction; however, in these motifs, the junctions were not migrating and were essentially immobile [1,3]. With sticky-end pairing, single building blocks—multi-arm junctions and diverse tiles consisting of a few adjacent DNA strands connected via crossovers [4]—could be assembled together, thus forming larger entities, such as lattices and nanotubes [5]. Without any exaggeration, it has been a rather rocky road from the dawn of DNA nanotechnology to its current enabled state [6,7,8]. Nevertheless, the rapid constantly cheapening synthesis of custom DNA sequences [9] and the development of new design methods [8]—in particular, the modular DNA origami technique [10,11,12,13], user-friendly computer-aided design software [14,15,16], and advanced simulation tools [17,18,19,20,21,22]—have all driven the evolution of structural DNA nanotechnology. As of today, programmable DNA nanostructures [23,24] may find uses in a variety of applications [23,24,25], ranging from materials science, nanofabrication, photonics, and microscopy [26,27,28,29,30,31,32] to robotics, therapeutics, and diagnostics [33,34,35,36,37,38].

It is reasonably safe to state—with no need to downplay other design techniques—that the introduction of DNA origami has enabled ever-increasing complexity in DNA shape space [6,8], and currently, it is the most routinely employed method among the DNA nanotechnology community [8]. Typical DNA origami nanostructures are folded from a ~7,000-nucleotide (nt) long M13 virus genome, i.e. a single-stranded DNA (ssDNA) scaffold, with the help of a unique set of staple strands [10]. These structures are usually at the megadalton scale and are designed using caDNAno [14,15], where the target shape is “sculpted” from square or honeycomb lattices that comprise of closely packed cylinders, which represent adjacent and parallel double-stranded DNA (dsDNA) domains. It is also possible to introduce twists [39] and curves [39,40] to DNA origami via rational design and approximate the outcome by using a finite-element based computational framework such as CanDo [17,18,19] or coarse-grained simulation software such as oxDNA [41,42]. Importantly, customized and relatively rigid DNA nanostructures can form micrometer-scale structures in the gigadalton regime through shape-complementarity [43] or algorithmic assembly [44]. DNA origami can also elicit mechanical movement, and these dynamic structures could be used, for example, as sensors, gates, or drug capsules [45,46].

However, there remain several drawbacks and restrictions in these general DNA origami methods. One obvious challenge in caDNAno-based design comes from its limitation to lattices, which makes formation of material-efficient, open, hollow or porous nanoscale conformations practically impossible. From another pragmatic point of view, structures having such conformations may be extremely feasible for many biomedical applications, as they apparently fold rapidly under distinct conditions and show better stability in biological buffers and at low-cation concentrations compared to lattice-based origami with closely packed helices [47,48,49].

**Figure 1:**
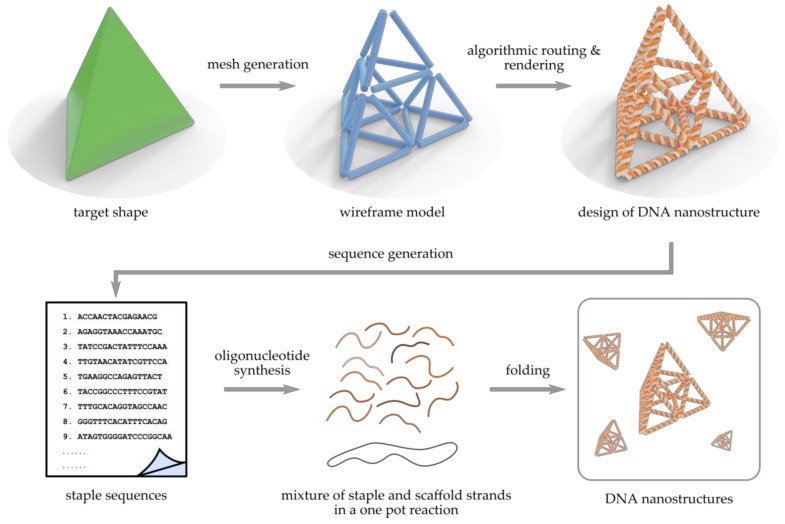
Pipeline for scaffolded wireframe DNA nanostructure production. Based on a continuous target shape (green tetrahedron), a discrete wireframe mesh (blue cylinders) is either designed manually or generated automatically. Each edge in the wireframe model can represent a single dsDNA, a double crossover (DX) molecule/two-helix bundle (2HB) or a 6-helix bundle (6HB) depending on the selected method. An approach-specific algorithm is then used to calculate the route of the single-stranded scaffold strand traversing the mesh and to render the blueprint of the DNA nanostructure (orange and gray). According to the routing, sequences of staple strands that are complementary to the known scaffold segments will be generated. Finally, the synthetic staples and the scaffold strand (circular or linear) solution are mixed and folded into billions of identical wireframe DNA nanostructures in a one pot reaction.

Therefore, during the past five years, the advanced wireframe-based construction of DNA structures has enjoyed rocketing attention [50]. Practically, it means that meshing and rendering—well-known methods in macroscopic engineering and computer graphics—are applied to nanoscale. The fundamental idea of wireframe DNA origami designing is described in Figure 1, and its general principles are explained in detail in Section 2. Briefly, the common workflow from a sketched target shape to a ready, folded wireframe DNA structure includes a formation of mesh of a target shape—a set of shape-defining vertices, edges, and faces—and the rendered wireframe model, followed by the scaffold strand routing through the meshes using a custom algorithm, and finally the generation of the sequences that staple the scaffold into the shape. Along with the expansion of the shape space and increasing complexity of the wireframe DNA objects, there has been a significant development in graphical design software. This evolution has led to new top-down paradigms that aim to reduce human input in the design process to minimum. After selecting the target shape, automatization of the full pipeline—meshing, rendering, strand routing, and sequence design—may remarkably lower the barrier for researchers (also outside the DNA nanotechnology field) to create their own two- and three-dimensional wireframe objects for user-specified purposes [50]. In this review, we shortly evoke the general wireframe-based design principles, discuss the recent progress in a nearly chronological manner, describe the currently available techniques and software, and summarize the demonstrated diverse wireframe DNA nanostructures. We believe this review will serve as a primer to wireframe DNA nanostructure design.

## 2. Wireframe Design Principles

Despite the most commonly harnessed DNA origami [10,11] being a lattice-based design motif, its precursor was in fact a wireframe nanostructure. The octahedron by Shih et al. [51] first demonstrated the cooperative assembly of a long ssDNA with the help of five 40-mer oligonucleotides. Before this “pre-origami” design, dozens of simple wireframe-based motifs, “meshes”, and structures assembled from a few strands, such as a cube [52], tetrahedra [53], polyhedra [54] and lattices with double crossover (DX) or two-helix bundle (2HB) edges, i.e. two parallel dsDNA molecules interconnected via crossovers [55], were demonstrated. Yet another approach for creating larger wireframe-like polyhedral objects is to employ rigid DNA origami tripods as building blocks [56]. These approaches are extensively reviewed in Ref. [57]. Here we focus on the recent, most flexible, and streamlined methods that are paving the way for the fully automated top-down design and fabrication of complex wireframe DNA shapes.

Much of the appeal of modern wireframe techniques is user-friendliness and generalization over different shapes [58]. The core idea for fabricating scaffolded wireframe DNA nanostructures in a top-down manner is visualized in Figure 1. First, the desired object is sketched using any graphical design tool. Then, in order to realize that object as a DNA nanostructure, straightforward semi-automated or fully automated pipelines can be followed without much need for manual user input. The key to this is turning the initial 2D or 3D model into a skeleton of itself, a wireframe mesh, that can function as a map for DNA strands. Depending on the approach, different algorithms are used to route a long DNA scaffold throughout the mesh in an appropriate way, so that each vertex and edge of the model is filled. It is then possible to assign a sufficiently long DNA sequence to the routed scaffold path and, similarly as in DNA origami techniques, generate short and complementary staple strands that will fold the scaffold into shape. The scaffold and staple sequences can then be exported from the design tool and synthesized. By folding these strands in a one pot reaction, large quantities of meshed DNA replicas of the initially modelled shape can be created in an easily accessible way.

The employed mesh routing is connected to graph theory and a Eulerian circuit pathing problem, where the goal is to systematically find an optimal path through the used network by only crossing each edge of the mesh once. This kind of routing can become very complex (NP-hard) depending on the used mesh size and pathing method, and it usually requires an automated algorithm to do it [59]. Different types of methods have been developed to approach this problem and these are discussed in their dedicated sections: gridiron and simple meshes (3.1.), semi-automated polyhedral rendering (3.2.), and fully automated design programs (3.3.).

In addition to the methods based on scaffold routing explained above, there also exist so-called scaffold-free approaches that omit the use of a long DNA scaffold altogether. An important benefit of this kind of approach is that they are not constrained by the lengths of available DNA scaffold strands and that they may appear more generalizable. This means designable structures are also not constrained in their size and scale unlike in scaffolded methods. Their design relies on modularity and the creation of small, interacting building blocks from DNA (individual strands, multi-arm junctions, tiles, etc.), that systematically compose into larger nanostructures. These approaches are presented and discussed in Section 3.4.

## 3. Shape Space, Design Strategies, and Software

### 3.1. Gridiron and Simple Meshes

**Figure 2:**
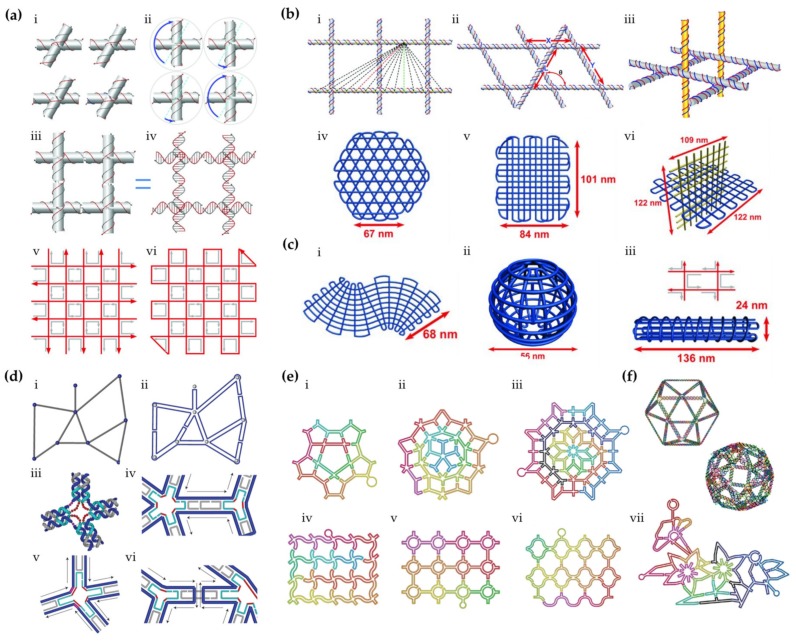
(**a**–**c**) DNA gridiron and (**d**–**f**) DNA origami with multi-arm junction vertices. (**a**) i: Relaxed conformations of different four-arm junctions. Note that the upper two junctions are rotated 180° in-plane with respect to the lower two. ii: Junctions rotated either 30° counterclockwise or 150° clockwise (blue arrows) to allow a gridiron unit formation. iii and iv: helical models illustrating a complete gridiron unit. v: scaffold directions in a simple 2D gridiron. vi: the zigzag pattern of scaffold and 90° rotation at corners to close the scaffold loop. (**b**) i: Possible connection points and directions for additional layers on a double-layer gridiron lattice, ii: angle calculation for a non-perpendicular lattice structure, iii: intertwining gridiron planes, iv: a three-layer hexagonal gridiron design, v: a four-layer gridiron design, vi: a 3D gridiron by intertwining planes. (**c**) Curved gridiron structures. i: S-shaped structure, ii: a sphere, iii: a screw. (**d**) Design principles of multi-arm junction structures. i: an arbitrary wireframe pattern composed of line segments (grey) and vertices (blue), ii: routing of the scaffold in 3 steps: 1. double the lines, 2. connect the lines that meet at vertices and 3. ‘loop’ and ‘bridge’ the segments into a continuous scaffold. iii,v: helical and line model of a four-arm junction with red segments representing additional poly-T for angle adjustment. iv,vi: typical staple strands routing examples. (**e**) Intricate 2D patterns with multi-arm junctions. i: a star-shaped pattern, ii: a Penrose tiling, iii: an eight-fold quasi-crystalline pattern, iv: a wavy grid, v: a circle array, vi: a fishnet, vii: a flower-and-bird pattern. (**f**) 3D wireframe Archimedean solid structures: a cuboctahedron and a snub cube with 24 vertices and 60 edges. (**a**–**c**) reproduced with permission from [60]. Copyright The American Association for the Advancement of Science, 2013. (**d**–**f**) reproduced with permission from [61]. Copyright Springer Nature Ltd., 2015.

A few years after the invention of 3D DNA origami, the idea of wireframe construction was revisited by the research group of Yan et al. [60]. They assembled gridiron-like DNA structures with increased complexity. Conceptually, the gridiron structure comprises of a series of four-arm junctions as vertices (Figure 2a, i–iv). In reality, the vertices are not physically disconnected tiles but intersected antiparallel scaffold lines in different layers (Figure 2a, iii–v). The scaffold, which forms the backbone of the gridiron structure, follows a zigzag path in the 2D design; in the most basic case, the first layer is routed in a zigzag manner and then rotated 90° in a corner, followed by another zigzag pattern in the second layer (Figure 2a, vi), thus forming the closed loop. The vertices are immobilized with the help of staples running in a circular path along the edges of every other opening (Figure 2a, v–vi). The shape space of the gridiron structure has also been expanded to multilayers, e.g., a hexagonal grid and three-dimensional grid as depicted in Figure 2b. By adjusting the arm lengths of the four arm junctions, curved 2D or 3D structures such as a sphere and a screw could also be created (Figure 2c).

In 2015, the same research group invented a more generalized design principle for arbitrarily shaped wireframe architectures [61] by introducing concepts in graph theory [62]. In this approach, vertices are not limited to four-arm junctions, which allows versatile angles between two edges (Figure 2d, i). Moreover, edges between vertices are constructed by 2HBs (DXs) instead of single dsDNA, therefore yielding more freedom for the circular scaffold to traverse all the vertices in the graph (Figure 2d, ii). Following the routing of the scaffold, staple strands are then mapped and engineered with crossovers holding the antiparallel edges together (Figure 2d, iii–vi). By adding poly-T loops with various lengths in the middle of junctions, the angles between adjacent branches can be determined (red segments in Figure 2d). With the design principle established, the authors demonstrated the potency of the method with remarkably complex structures, which include 2D objects like Penrose tiles, curved, and circular patterns and even an abstracted drawing with a hummingbird and flowers (Figure 2e) as well as 3D Archimedean solids (Figure 2f). As a follow-up, Yan’s group showed that using layered crossovers, i.e. crossover pairs that connect neighboring layers of DNA duplexes, the relative orientation of the layers can be adjusted in the multilayer wireframe designs and thus create arbitrary 3D frameworks [63]. The structures and their integrity in Refs. [60,61,63] were verified by transmission electron microscopy (TEM), cryo-electron microscopy (cryo-EM), and atomic force microscopy (AFM).

The abovementioned wireframe architectures were designed using a software called Tiamat [64,65], which is a general-purpose editing tool for any 2D or 3D DNA structure with sequence generation function. However, users still need to apply their own routing algorithm, either manually or with customized scripts, before inputting the design into Tiamat. Therefore, it is not quite user-friendly, especially for structures with increasing complexity. Besides designing, thorough validation and analysis of the wireframe nanostructure before the actual production is essential for avoiding the waste of resources due to design errors. An alternative lattice-free CanDo simulation mode developed by Pan et al. [66] could serve the purpose; however, it is limited to structures with four-arm junction vertices.

There also exist other methods for wireframe structures with simple meshes. For example, Matthies et al. [67] demonstrated a DNA truss with triangulated mesh. Later, Agarwal et al. [68] from the same research group showed that DNA polymerase can be employed to fill the single-stranded gaps in a truss structure with dsDNA after the assembly owing to the better accessibility compared to lattice-based DNA origami with closely packed helices. The triangulated meshes in both of these works were realized using a custom-developed script called “k-route” [67].

Up to this point, the strategies for building wireframe DNA origami were still largely dependent on user input. To further increase the complexity and functionality of wireframe designs, it is essential to introduce semi-automatic and automatic tools to researchers with different backgrounds. In the following subsections, such tools will be discussed in more detail.

### 3.2. Semi-Automatic Top-Down Polyhedral DNA Rendering with vHelix

**Figure 3:**
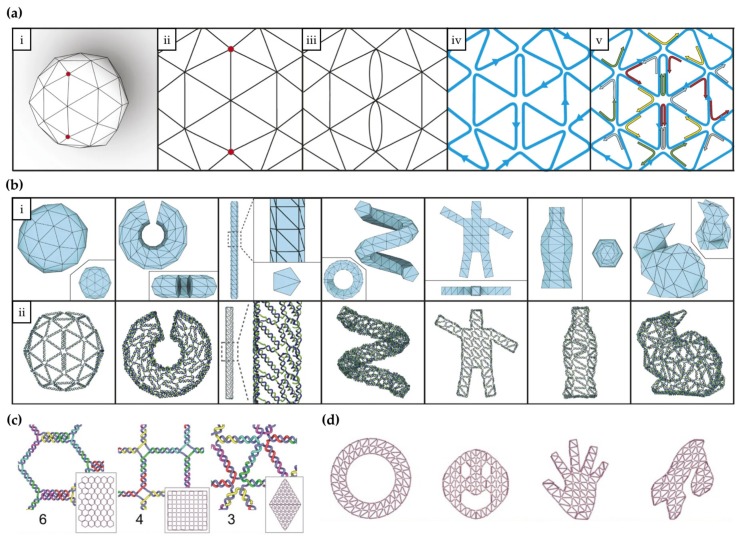
(**a**–**b**) Semi-automated DNA rendering of polyhedral meshes and (**c**–**d**) flat sheet meshing using vHelix. (**a**) Scaffold routing and sequence design for a scaffolded object with spherical topology. i: A meshed target shape. ii: The triangular meshwork forms a Eulerian circuit of edges connected by vertices. iii: The solution for optimal (A-trail) routing may require passing the route through a minimal number of edges twice. iv: A single-stranded DNA scaffold is systematically routed throughout the mesh by a routing algorithm. v: A sequence is applied to the scaffold and complementary staple strands are generated for folding the structure into a desired shape. By exporting, synthesizing, and folding the used strands, the target shape can be formed from DNA. (**b**) Models of various, increasingly complex DNA nanostructures created with vHelix. i: Used polyhedral mesh models. ii: Corresponding models with scaffold routing. From left to right: A sphere, a nicked toroid, a rod, a helix, a waving stickman, a bottle, and a Stanford bunny. (**c**) The different tessellation meshworks usable for 2D wireframe objects. From left to right: A hexagonal, a square, and a triangular mesh. (**d**) Models of various 2D shapes created with vHelix. From left to right: A ring, a face, a hand, and a map of Norway, Sweden and Finland. (**a**–**b**) reproduced with permission from [69]. Copyright Springer Nature Ltd., 2015. (**c**–**d**) reproduced with permission from [73]. Published by John Wiley & Sons, 2016.

An intuitive top-down design and modeling tool for wireframe DNA replicas of user-defined 2D and 3D objects is provided by the recent vHelix technique developed by the research group of Högberg et al. [69]. vHelix is a toolkit plugin for the CAD program Autodesk Maya [70]. In their approach, a 2D or 3D model of desired shape is first created in Autodesk Maya and that model is then made into a triangulated, polyhedral wireframe mesh with any applicable meshing tool (Figure 3a, i). This polyhedral model is then processed with the BSCOR software package, where a single-stranded DNA scaffold is routed along the edges of the mesh in an automated way by an algorithm. The routing of the scaffold through triangulated meshes is the so-called Chinese postman problem, for which an Eulerian circuit, an A-trail, is the optimal solution; the scaffold passes each edge of the mesh only once, and without crossing straight over any vertices [71] (Figure 3a, iv). However, depending on the mesh, this kind of routing is not always possible, and therefore, the solution of this optimization problem is to introduce a minimal amount of duplicate “helper” edges (scaffold passing twice through these edges) (Figure 3a, iii–iv). To improve the structural soundness of these wireframe structures, the routed models can be mechanically modeled as rods interconnected by springs with a physical modeling tool such as NVIDIA PhysX [69]. The design can then be incrementally adjusted to relax any detrimental strains in the DNA mesh and reloaded back into vHelix for further manual adjustment and finalization. Next, the single-stranded edges are supplemented with short, complementary staple strands to make the structure robust (Figure 3a, v). By defining the scaffold sequence, the staple sequences can be exported, synthesized, and eventually folded with scaffold strand to form wireframe DNA nanostructure from the design. A variety of objects with increasing complexity has been designed with vHelix in this way from a simple sphere to the wireframe Stanford bunny (Figure 3b) and verified using TEM imaging.

A study on the effects of design choices on the stiffness of these wireframe structures was undertaken by the same research group in 2018 [72]. Through physical simulation with oxDNA software [41], synthesis, and TEM and AFM characterization of various test DNA meshes, they connected DNA wireframe stiffness with the persistence lengths of constituent double-helices and the salt concentrations of buffer solutions. They simulated monovalent salt conditions in a range of 100–735 mM (Na^+^) and found that lower concentrations of the monovalent cations help DNA helices retain internal repulsion and thus maximize persistence length and overall stiffness. Importantly, having the structures in the lower, physiological salt concentrations (150 mM Na^+^, low Mg^2+^) also brings them closer to real-life applications.

While a seemingly versatile and semi-automated method, there still exists a constraint that the conceivable 3D shapes need to be inflatable to a ball—in other words, the shapes need to have spherical topology—and to make routing with the original vHelix protocol possible [69]. However, by implementing a modified routing algorithm, the group was able to create a new pipeline applicable for 2D objects and regular tessellation meshes (hexagons, squares and triangle lattices) [73]. In this newer strategy, a larger rectangular sheet of polygonal mesh is created first and the desired object is then sculpted by omitting, moving and re-scaling vertices and edges in the sheet. Using this, the group created a set of structures with the three different polygonal meshworks (Figure 3c) and found the triangular meshwork resulted in the best folding yield and strength for their designs. As with the original 3D vHelix approach, this 2D modification allows for high complexity and freedom in creatable shapes (Figure 3d). Although not demonstrated, this 2D method might also be possible to be applied with a mixed polygonal mesh, creating regions of variable malleability in a ready object.

### 3.3. Automatic Top-Down Fabrication with DAEDALUS, PERDIX, TALOS, METIS, and ATHENA

**Figure 4:**
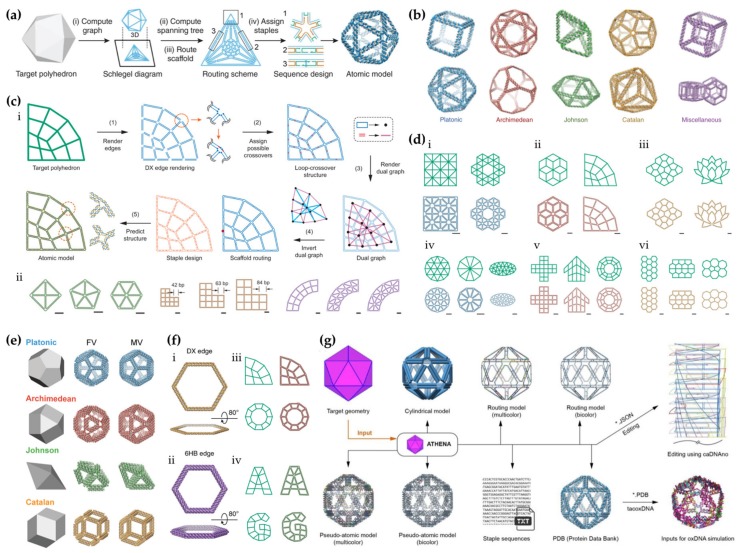
(**a**–**b**) Automated DNA origami design with DX edges using DAEDALUS, (**c**–**d**) autonomously designed free-form 2D origami using PERDIX, (**e**) automated 3D DNA origami design with honeycomb edges using TALOS, (**f**) automated 2D DNA origami design with DX and honeycomb edges using METIS and (**g**) DNA origami design process using ATHENA. (**a**) DAEDALUS workflow; a 3D graph (step i) and the spanning tree (step ii) are computed for the meshed target shape, followed by the scaffold routing by the spanning tree algorithm with an Eulerian circuit (step iii) and sequence design (step iv) with a predicted atomic model. (**b**) Examples of Platonic, Archimedean, Johnson, and Catalan solids and miscellaneous shapes created by DAEDALUS. (**c**) i: PERDIX workflow; a 2D graph with meshes and corresponding DX-edges are rendered for the target object (step 1) in order to generate the loop-crossover structure (step 2) and to enable assigning crossovers by computation of node-edge dual graph (step 3) followed by the scaffold routing through the whole object (step 4) and the assignment of complementary staple strands and a prediction of atomic model (step 5). ii: Designed objects with variable vertex degrees, edge lengths, and internal meshes. (**d**) Versatile object geometry with internal triangular (i,iv), quadrilateral (ii,v) and N-polygonal (iii,vi) mesh objects. The scale bars in (**c**–**d**) are 20 nm. (**e**) Examples of Platonic, Archimedean, Johnson, and Catalan solids with flat vertex (FV) and mitered vertex (MV) designs using TALOS. (**f**) i–ii: DX- and 6HB-edge DNA origami hexagons without internal mesh. iii: Quadrilateral mesh objects with 6HB-edges. iv: Letter-shaped wireframe objects with irregular mesh using METIS. (**g**) ATHENA software; The software performs scaffold routing and defines staple sequences automatically. It also produces several models for computer simulations and allows external editing of staples in caDNAno software. (a–b) reproduced with permission from [50]. Published by Springer Nature Ltd., 2016. Original figures reproduced with permission from [74]. Copyright The American Association for the Advancement of Science, 2016. (**c**–**d**) reproduced with permission from [76]. Published by The American Association for the Advancement of Science, 2019. (**e**) reproduced with permission from [78]. Copyright American Chemical Society, 2019. (**f**) reproduced with permission from [82]. Published by Springer Nature Ltd., 2019. (**g**) reproduced with permission from [84]. Copyright by the authors of [84], 2020.

Soon after the introduction of the ingenious vHelix software, the research group of Bathe et al. solved some of the flaws in the top-down approach and upgraded the process from semi-automatic to completely automatic. First, they introduced DAEDALUS (DNA Origami Sequence Design Algorithm for User-defined Structures) [74,75], a fully automated spanning-tree algorithmic framework that enables the top-down wireframe design and fabrication of 3D objects virtually in any shape. In all, 45 complex nanoparticle geometries were produced according to the scheme depicted in Figure 4a, including Platonic, Archimedean, Johnson, and Catalan solids and several asymmetric constructs and polyhedra with non-spherical topologies (Figure 4b). A facile asymmetric polymerase chain reaction (aPCR) was employed to obtain custom-length linear scaffold strands, shorter (450 to 3,400 nt) or longer than the ordinary M13 phage DNA (7,249 nt), beneficial to minimize the excess of single-stranded DNA and thus, improving folding yields.

A few years later, the group developed another algorithmic approach dubbed PERDIX (Programmed Eulerian Routing for DNA Design using X-overs) [76,77], in order to address the shortcomings of DAEDALUS software on rendering 2D wireframe assemblies. The open-source program is based on an automatic procedure that allow 2D free-form geometry design with the internal mesh geometry rendered automatically by the algorithm that also performs automatic scaffold and staple routings, converting each edge into two parallel DNA duplex edges of arbitrary length based on antiparallel DX crossovers with multi-arm junctions at variable vertices. Another option is fully autonomous DNA rendering specifying the complete internal and external boundary geometry in the final origami object (Figure 4c, i). A minimum edge length of 38 bp was required to obtain at least two double crossovers per edge. Utility and robustness of the assemblies were demonstrated by designing variable vertex degree (2–6), edge lengths (42, 63, and 84 base pairs (bp)), and internal mesh (Figure 4c, ii), with triangular, quadrilateral, and N-sided polygonal mesh patterns, respectively (Figure 4d). The structural fidelity of all 2D origami objects was verified by AFM imaging.

The group introduced yet another open-source software TALOS (Three-dimensional, Algorithmically generated Library of DNA Origami Shapes) [78,79] that broadens the scope of the 2D and 3D wireframe sequence design procedures by employing 6HB motifs instead of dsDNA or DX molecules as edges, which should enhance mechanical stiffness, biological stability, and resistance against the nucleases [47,80,81]. The TALOS algorithm enabled the automated design of 3D polyhedra consisting of edges of any cross section with an even number of duplexes and subsequent utilization to DNA structures composed uniformly of single honeycomb edges. In addition to the “flat vertex” (FV) motif, i.e. a single-vertex scaffold crossover between each pair of neighboring edges, a new three-way vertex crossover, named as “mitered vertex” (MV) motif, was introduced. The elaborated design enabled the construction of shapes with variable edge lengths and vertex angles and thus, realization of a highly asymmetric origami objects, structures otherwise inaccessible via DAEDALUS (see above) or vHelix-BSCOR (see Section 3.2.). 3D structural characterization was corroborated via agarose gel mobility shift assays, TEM, cryo-EM, and 3D reconstruction. The acid test for TALOS algorithm was applied when authors fabricated in silico 240 different polyhedral objects with sequence designs for all Platonic, Archimedean, Johnson, and Catalan solids, respectively (Figure 4e).

In addition to PERDIX and TALOS, an algorithm METIS (Mechanically Enhanced and Three-layered origami Structure) [82,83] was developed to enhance mechanical stiffness and fidelity of vertex angles in 2D wireframe origami. This is achieved by combining the above-mentioned methods; now the requirement of full turn (10.5 bp) of double helix at the design edges is not necessary. PERDIX generates target objects having varying vertex types with single-layer/DX-based wireframe motifs with or without internal mesh geometry (Figure 4f, i). In turn, METIS generates lattice-based geometries by stacking three layers corresponding to a cross-section of the six-helix bundle (Figure 4f, ii). Layers are connected by a three-way vertex crossover motif in which each duplex in a single-layer is connected to another duplex in the same layer in a neighboring wireframe edge, without the geometric limitations facilitated by the TALOS software package. Universality of this automated sequence design procedure was demonstrated by generating various lattices with 6HB edges (e.g. curved beam and star), objects without meshes (e.g. triangle, octagon), triangular- and quadrilateral (Figure 4f, iii) mesh objects, and irregular letter-like mesh object (Figure 4f, iv). Uniformity and structural fidelity of folded geometries were confirmed by AFM, TEM, and molecular dynamics simulations.

All these distinct algorithms are embedded in a software package ATHENA [84,85] that has an intuitive graphical interface, thus allowing the user to design any kind of 2D or 3D wireframe structure with 2HB or 6HB edges (Figure 4g). The scaffold routing and staple sequences are defined automatically for the target shape, and importantly, external editing of sequences using most commonly employed caDNAno software [14,15] is enabled in the workflow. Therefore, the process facilitates trouble-free modifications to the object and it allows for the position of other molecular components, such as nanoparticles or drugs, to the structure with high precision and addressability. The software also produces atomic-level models for molecular dynamics and coarse-grained dynamics simulations that help to verify the shape, stiffness, and structural details before folding.

### 3.4. Scaffold-Free Approaches

So far, we have only discussed techniques that rely on scaffolded design. However, there exist design strategies that are, in principle, more generalizable as they are essentially scaffold-free. This means the structures are again constructed from the predefined building blocks that can be individual strands, (multi-arm) junctions or other simple motifs. Wei et al. [86] propelled a shift in the conventional design paradigm by omitting the process of folding long ssDNA strands. Instead, they used modular rectangular DNA tiles with sticky ends to compose complex 2D canvases pixel by pixel. Ke and co-workers [87] evolved the concept and created even more versatile 3D canvases using sequence-specific 32-nt LEGO-like DNA bricks (4 × 8 bp binding domains) [88] (CAD models can be converted to DNA brick structures using LegoGen [89]). Ong et al. [90] elaborated the concept further by increasing the “voxel” size to 52 nt (4 × 13 bp domains) and 74 nt (2 × 18 bp and 2 × 19 bp domains), which enhanced both the kinetics of the assembly process and yield due to the larger sequence space. This allowed the construction of up to gigadalton sized cuboids with 30,000 components. Authors developed the Nanobricks software tool [91] to enable sculpting DNA brick cuboids to mathematically complex cavities and other 3D objects.

**Figure 5:**
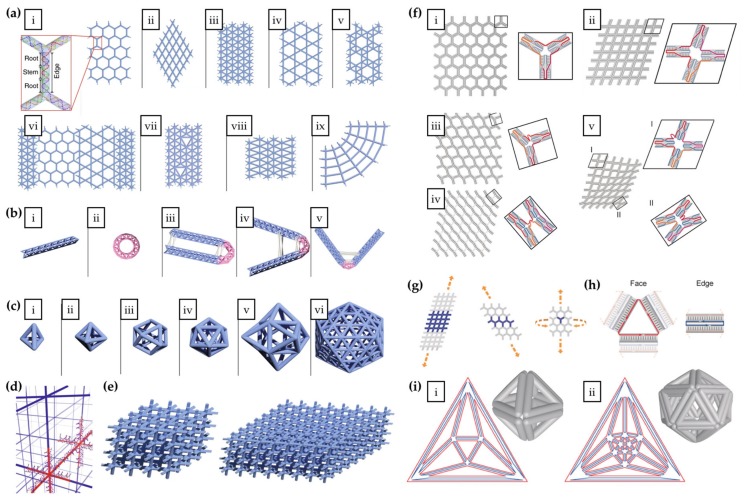
(**a**–**e**) Scaffold-free complex wireframe structures from simple building blocks and (**f**–**i**) wireframe designs from junction motifs. (**a**) i: A zoom-in view of complementary root-stem base pairs in the edge formation. i–ix: Illustration of an increasing complexity of 2D tessellation patterns. (**b**) Wireframe tubes with 6-arm vertices showing diagrams of straight tube (i), donut (ii), U-bent (180°-bent) (iii), 135°-bent (iv), and 90°-bent (v) tubes. (**c**) Wireframe polyhedra illustrating a tetrahedron with 3-arm vertices (i), an octahedron (ii), a cuboctahedron with 4-arm vertices (iii), an icosahedron with 5-arm vertices (iv), a triangulated cube with 6-arm vertices (v), and a triangulated Buckyball with 5-arm and 6-arm vertices (vi). (**d**) DNA duplexes (shown in red) overlaid on a grid. (**e**) Fully addressable 3D arrays i.e. “nanocrystals”; a 4 × 4 × 4 array (left) and an 8 × 8 × 4 array (right). (**f**) Scaffold-free 2D nanostructures from junction motifs. i: a honeycomb grid with Y-shaped (3-arm) motifs. ii: a rhombic grid with X-shaped (4-arm) motifs. iii–v: 2D wireframe structures with Y- and X-shaped motifs showing angle control. (**g**) Extended ribbon structures from X- and Y- motifs and tubular structure from Y-motif. (**h**) Illustration of the representative face and edge in a 3D polyhedron. (**i**) Schlegel diagrams (colored) and cylinder models (grey) of a DNA octahedron (left) and a DNA icosahedron (right). (**a**–**e**) reproduced with permission from [92]. Published by Springer Nature Ltd., 2019. (**f**–**i**) reproduced with permission from [93]. Copyright John Wiley & Sons, 2019.

The design program developed by Wang et al. [92] enables the fabrication of scaffold-free wireframe structures composed of a predesigned ratio of node-edge network glued together with a complementary root-stem domain base pairs. Each node represents vertices with 3–6 arms, whereas edges are simple DNA duplexes with various lengths. This highly versatile self-assembly method allows increasing complexity of the structures ranging from various 2D tessellation patterns (Figure 5a) to a toroid and a plethora of wire-frame tubes with different bending angles (Figure 5b), polyhedra (Figure 5c), and ultimately to fully addressable 3D multilayer nanocrystal arrays (Figure 5d) with different lattice geometries (Figure 5e). The scaffold-free approach provides more flexibility in design and morphology than the techniques relying on predefined discrete scaffolds. This approach facilitates the construction of megadalton-sized structures with high material efficiency and rigidity, specifically via triangulation connectivity. Furthermore, the authors also point out that the inherent porosity of the designs endows hosting of various bioactive cargo.

Huang et al. [93] improved the scaffold-free LEGO approach by bundling two duplexes by crossovers on both ends (crossovers at vertices) rather than bundling crossovers in the middle of the helices as before. Both Y- and X-shaped motifs, each arm possessing 52-nt (4 × 13 bp duplex) strands, were designed to furnish addressable honeycomb and rhombic grids (Figure 5f, i–ii). Instead of the assumed square shape, self-assembly of X-motifs (4-arms) generated a rhombic shape, probably due to minimization of electrostatic repulsions at the backbone and maximization of adjacent base stacking at the vertices. Vertex angles were manipulated by inserting one or two 10-bp duplexes at the crossover points leading to T-shaped and cross-shaped vertices (not shown) and other new 2D wireframe junctions (Figure 5f, iii–v). Extended 1D wireframe lattices from Y- and X-motifs (Figure 5g, left and middle), and a 2D tubular configuration (Y-motif) (Figure 5g, right) were obtained using 16 nt domain manifolds. Construction of polyhedral—an octahedron (Figure 5i, i) and an icosahedron (Figure 5i, ii)—required a new design for complementary bundled DNA duplexes (Figure 5h), a triangle (face with 3 nicks), and a rectangle (edge with 2 nicks), due to 5′-to-3′ polarity of DNA strands. Authors postulate that the marriage of both techniques paves the way for economical production of precise nanostructures of high complexity.

## 4. Conclusions and Future Perspectives

The rapid development of user-friendly methods to create designer DNA nanostructures has remarkably lowered the barriers to real-life applications. One of the key elements in this progress is that the emerging cost-efficient techniques are gaining increasing amounts of attention from people outside the DNA nanotechnology field—both from academia and industry. The transformation of any desirable shape visualized on the computer screen into billions of real objects in a test tube is straightforward and fast, owing to the sophisticated CAD software for each distinct DNA design paradigm. As described above, all the algorithms for automated top-down design methods developed by Bathe et al. are now integrated into one graphical software ATHENA [84,85]. In addition, the modeling and visualization software Adenita by Barišić and co-workers provides a way to combine lattice-based design to wireframe constructions, thus also allowing hybrid structure designs [16]. These kinds of interfaces will definitely help both experts and non-specialists to create their own DNA nano-objects. Importantly, the research group of Bathe et al. has already shown that their designer wireframe DNA origami shapes may have an imminent biomedical application, as they created various precise multivalent arrangements of a clinically-relevant HIV gp120 immunogen on the virus-like DNA particles to systematically probe their impact on B cell triggering in vitro [94]. We strongly believe that all these versatile structures will find uses not only in biological research but also in assembling novel functional materials [95,96,97,98].

However, some obstacles and open questions still remain on the way toward revolutionizing implementations, especially in biomedicine [38,99]. The stability of the wireframe structures seems to be rather different compared to the DNA structures designed in particular on 3D lattices [50,69,74], but interestingly, there are also reports suggesting that 2D DNA origami or rolled sheets may survive in physiological conditions for several hours [100,101]. Some wireframe shapes can be folded in low-magnesium or low-sodium conditions, and they have also elicited better stability in biological cell-compatible buffers, such as phosphate buffered saline, when contrasted with their lattice-based companions [50,69,74]. The other important issues are their partially unknown sequence—and superstructure-dependent drug-loading properties [38,102]—as well as susceptibility to nucleases [103,104]. Unfortunately, it is extremely hard to make comparisons between different design strategies and DNA shapes as the conditions vary between each reported study. Even so, it seems that as a result of the modularity of the wireframe scaffold-free objects, their nuclease digestion profiles could be rationally designed, as demonstrated by Wang et al. [92]. This may be extremely interesting in for example sequential release of loaded biomedical cargoes from the DNA vehicles. Nevertheless, there are several ways to increase the overall durability, biocompatibility, and bioavailability of the DNA shapes using protective polymer-, lipid-, protein- and peptoid-coatings [81,105,106,107,108,109,110,111,112], cross-linking of the DNA strands [113] or the DNA-coating polymers [114]. These methods have often been demonstrated for lattice-based designs, but some of them are equally available for wireframe structures [107]. Therefore, the combination of presented automated design methods with biotechnological mass production of DNA [9], various application-specific protection mechanisms, and the scaling-up capabilities of the assemblies [115,116,117,118] will undoubtedly pave the way for a plethora of applications and for the full commercialization of ready-to-use DNA nanostructure fabrication based on researcher/customer needs [7,119].
